# A Cognitively-Motivated Framework for Partial Face Recognition in Unconstrained Scenarios

**DOI:** 10.3390/s150101903

**Published:** 2015-01-16

**Authors:** João C. Monteiro, Jaime S. Cardoso

**Affiliations:** INESC TEC and Faculdade de Engenharia, Universidade do Porto, Campus da FEUP, Rua Dr. Roberto Frias, n 378, 4200-465 Porto, Portugal; E-Mail: jaime.cardoso@inescporto.pt

**Keywords:** biometrics, face recognition, partial data, universal background model, Gaussian mixture models

## Abstract

Humans perform and rely on face recognition routinely and effortlessly throughout their daily lives. Multiple works in recent years have sought to replicate this process in a robust and automatic way. However, it is known that the performance of face recognition algorithms is severely compromised in non-ideal image acquisition scenarios. In an attempt to deal with conditions, such as occlusion and heterogeneous illumination, we propose a new approach motivated by the global precedent hypothesis of the human brain's cognitive mechanisms of perception. An automatic modeling of SIFT keypoint descriptors using a Gaussian mixture model (GMM)-based universal background model method is proposed. A decision is, then, made in an innovative hierarchical sense, with holistic information gaining precedence over a more detailed local analysis. The algorithm was tested on the ORL, ARand Extended Yale B Face databases and presented state-of-the-art performance for a variety of experimental setups.

## 1. Introduction

Personal identification plays an important role in almost everyone's daily activities. Knowledge-based and token-based automatic personal identification are the most used techniques to tackle this problem. Token-based approaches take advantage of a personal item to distinguish between individuals, whereas knowledge-based approaches are based on something the user knows to which, theoretically, nobody else has access [1].

Both of these approaches present obvious disadvantages: tokens may be lost, stolen or forgotten, while passwords can easily be forgotten by a valid user or guessed by an unauthorized one [2]. In fact, one can summarize the problem of these approaches by pointing that any piece of material or knowledge can be fraudulently acquired.

Biometrics can be seen as a return to a more natural way of identification. By attempting identification based on physiological and behavioral traits, we are testing someone by who (s)he is, instead of relying on something (s)he owns or knows. Such an approach seems likely to be the way forward [3].

Over the past few years, the issue of face recognition has been in the spotlight of many research works in pattern recognition, due to its wide array of real-world applications. The face is a natural, easily acquirable and usable trait with a high degree of uniqueness, representing one of the main sources of information during human interaction [4]. These marked advantages, however, fall short when images of limited quality, acquired under unconstrained environments, are presented to the system.

It has been noted that the performance of face recognition algorithms is severely compromised when dealing with non-ideal scenarios, such as non-uniform illumination, pose variations, occlusions, expression changes and radical appearance changes [5]. Whereas technological improvements in image capturing and transmitting equipment managed to attenuate most noise factors, partial face occlusions still pose a genuine challenge to automated face recognition [6].

Facial occlusions may occur due to a multiplicity of deliberate or unintentional reasons. Whereas accessories, such as sunglasses and scarves, and facial hair represent quite common sources of occlusion in daily life, they can also be explored by bank robbers and shop thieves in an attempt to avoid recognition. Furthermore, the use of some accessories might be enforced in restricted environments (such as medical masks in hospitals and protection helmets in construction areas) or by religious or cultural constraints [5]. The fact that humans perform and rely on face recognition routinely and effortlessly throughout their daily lives leads to an increased interest in replicating this process in an automated way, even when such limitations are known to frequently occur [7].

Even though there is no consensus in the cognitive science field as to how the human brain recognizes faces, either based on their individual local features or, more holistically, on the basis of their overall shape [8], several works have shown that both levels of information play a non-negligible role in human face perception [9,10]. Whereas holistic representations provide a global summary of the spatial arrangement of contours and textures in an image, local features provide a more detailed regional description of the parts that compose it [11].

In the present work, we propose a robust alternative to face recognition under partial occlusions and variable illumination. An innovative hierarchical decision framework, incorporating both holistic and local descriptions, is proposed. The global precedent hypothesis for human perception [12] is the basis of this new decision strategy. Such a hypothesis claims that face recognition is performed by the human brain in a global-to-local flow, with holistic information gaining precedence over a more detailed local analysis. By following this rationale, we aim to replicate the cognitive process of face recognition by the human brain in an automated way. We evaluate the proposed algorithm on three widely-studied databases—the ORL, ARand the Extended Yale B databases—characterized by a variety of occlusions, small pose variations, facial expressions and illumination conditions.

The rest of the paper is organized as follows: Section 2 summarizes the most recent trends of research in the field of unconstrained face recognition. Section 3 outlines and motivates the proposed algorithm. Section 4 summarizes the most relevant experimental results obtained, as well as a detailed analysis and comparison with the state-of-the-art, and finally, in Section 5, we present the conclusions of the present work, as well as some suggestions for future improvements.

## 2. Related Work

Face recognition has been a widely-studied research topic in the last few decades. Some traditional approaches, like eigenfaces [13], Fisherfaces [14] and active appearance models [15], have become highly popular and laid out the foundations for a variety of commercial off-the-shelf systems. However, all of the previously mentioned techniques stumble upon the limitations presented in the last section: when non-ideal conditions are present during the acquisition step, recognition performance is severely compromised. The need to improve the state-of-the-art in face recognition to encompass a set of more realistic applications has, thus, been catalyzing research in the area to a set of new directions.

The study of invariant features to diminish the effects of occlusion, illumination and other nefarious sources of noise represents one of the most significant focuses of recent research. Liao *et al.* [16] tried to overcome the need for face alignment that characterizes most holistic approaches employing a multi-keypoint descriptor representation. In this work, any type of face image, either holistic or partial, can be probed for recognition, regardless of the global content. Nallammal and Radha [17] propose a non-negative matrix factorization (NMF) variation to explore the potential of the eyes and the bottom face regions for recognition when the probe images present a high degree of occlusion. An alternative approach is followed by Oh *et al.* [18], where random horizontal and vertical patches of face images are used as templates for cancelable identity verification. Such a technique intentionally distorts biometric information, in a repeatable, but non-reversible manner, to better deal with the compromising of biometric templates. Karande and Talbar [7] address the problem of face recognition with large rotation angles and variable illumination conditions through the use of edge information for independent component analysis (ICA). The work by Geng and Jiang [19] explores some of the known limitations of the widely-studied SIFT approach, for a specialized application in face image description. An alternative keypoint detection and a partial descriptor are both proposed in an attempt to adapt the traditional algorithm to non-rigid and smooth objects. Cho *et al.* [11] propose a two-step approach to face recognition, with principal component analysis (PCA) used at a coarser level and Gabor filtering at a finer level. This finer analysis in only carried out if the coarser recognition results do not present a high degree of reliability. Recently, Facebook's Deep Face Project claimed 97.25% accuracy, where humans achieve an accuracy of 97.53%. Their approach, published by Taigman *et al.* [20], was based on deep neural networks, allowing the effective use of highly complex statistical models trained for large volumes of data.

Recently, approaches based on sparse representation classification (SRC) have shown impressive performance in unconstrained face recognition and became one of the hot research topics in the area. The first reported use of SRC for face recognition, by Wright *et al.* [21], approached the problem of partial occlusion by representing face images as a linear combination of the whole face gallery and a vector of residuals at the pixel level. Classification was then achieved by *l*_1_ minimization of the vector of residuals for each possible identity. Zhou *et al.* [22] further improved this methodology by enforcing spatial coherence of occluded pixels through the use of Markov random fields (MRF). The spatial continuity of occlusions in face images was also explored by Qian *et al.* [23]. Their methodology takes advantage of the low-rank error images that are originated in occluded images by traditional SRC methodologies, to perform effective and robust recognition when such conditions are observed. Besides spatial coherence of occlusions, some other topics have been explored in recent works as possible improvements to the original SRC proposal. Wang *et al.* [24] propose an Adaptive SRC (ASRC) approach capable of selecting the most discriminative samples for each representation, using joint information from both sparsity (*l*_1_ minimization), as well as correlation (*l*_2_ minimization). Shen *et al.* [25] propose a variation of SRC for implementation in Android and iOS mobile devices. Their proposal optimizes the computation of residual values with significant gain in computational efficiency and no considerable losses in recognition accuracy. Jian *et al.* [26] also center their attention on the computational speed limitations of the SRC approach. Their proposal, based on the orthogonal matching pursuit (OMP) algorithm, achieves fast and robust face recognition, even though the best results are only achieved through a preliminary occlusion detection block. Even though considerable work has been performed in the area, the main drawback regarding the SRC approach is still posed by the need for an extensive and diverse library of well-aligned face examples.

Another focus of research in recent years concerns the use of prior knowledge regarding occlusions in face images. Zhang *et al.* [27] proposed an estimation of the probability distribution of occlusions in feature space using the Kullback–Leibler divergence (KLD). In a mixed approach regarding both previous detection of occlusions and SRC face recognition, Li *et al.* [6] present a two-step SRC approach, where SRC is both used to first discriminate occluded pixels from unoccluded regions and then to perform face recognition. The use of downsampled images allows a significant improvement in processing speed. Min *et al.* [5], on the other hand, perform occlusion detection using MRF to promote the spatial coherence of the detected occlusion regions. The recognition step is then carried out solely on the non-occluded regions. Even though the *a priori* detection of occlusions may significantly improve the accuracy of local face recognition, the introduction of a new block in the recognition system may bring about a new set of problems. An increase in the computational cost of the process, as well as the creation of a new source for errors that may condition the recognition process from its earlier steps can be counted among such challenges.

In the present work, we propose a robust approach to face recognition when non-ideal conditions, such as partial occlusions and severe illumination variations, affect the acquisition environment of the system. In an attempt to tackle most of the limitations presented in the works outlined in the last paragraphs, we designed a new hierarchical recognition framework. This innovative approach allows a considerable reduction in the computational cost of the whole recognition process, while also allowing an intuitive integration of multiple region-based details. The proposed algorithm is able to achieve accurate face recognition, even when a limited set of images with small variations is used for model training.

### 2.1. Algorithm Overview

**Figure 1.**
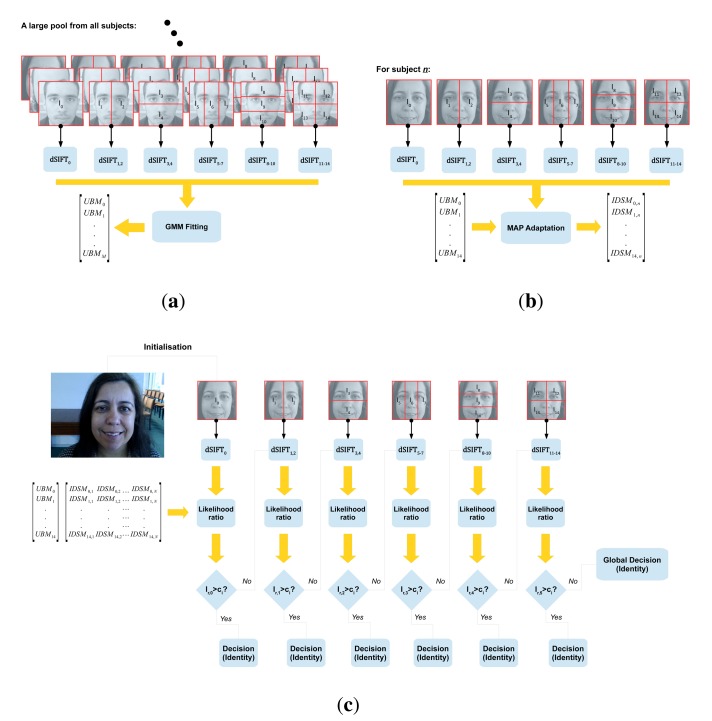
Schematic representation of the proposed algorithm and its main blocks: (**a**) training of the universal background models using data from multiple individuals; (**b**) maximum *a posteriori* (MAP) adaptation of the universal background models (UBM) to generate individual specific models; and (**c**) testing with new data from unknown sources.

The proposed algorithm is schematically represented in Figure 1. Figure 1a,b depicts the enrollment process in the proposed approach. During enrollment, a new individual's biometric data are inserted into a previously existent database of individuals. In the present work, a hierarchical ensemble of i partial face models is trained for each enrolled subject. The *M* individual-specific models are built by maximum *a posteriori* (MAP) adaptation of the corresponding set of *M* universal background models (UBM) using individual-specific data. The UBM is a representation of the distribution that a biometric trait presents in the universe of all individuals. MAP adaptation works as a specialization of the UBM based on each individual's biometric data. The idea of MAP adaptation of the UBM was first proposed by Reynolds [28], for speaker verification, and will be further motivated in the following sections.

## 3. Proposed Methodology

The database is probed during the recognition process to assess either the validity of an identity claim (verification) or the *k* most probable identities (identification) given an unknown sample of biometric data. In the present work, we propose an innovative approach to the recognition process based on the global precedence hypothesis of face perception by the human brain. Recognition is performed hierarchically, as depicted in Figure 1c, with global models taking precedence over more detailed ones. Partial models are hierarchically organized into levels. Each level is composed by a set of non-superimposing subregions, *I_l_*, of equal size (Levels 2–3 and 4–5 were hierarchically ordered in an arbitrary order, even though their composing regions are of equal size. Previous knowledge of expected types of occlusion could be explored when specifying this order.). Subregions at the same level sum to the full-face image, *I*_0_. During recognition, a test image from an unknown source follows the hierarchical flow depicted in Figure 1c, until a decision can be made with a significant degree of certainty. The significance of a decision carried out at a single level is defined through the analysis of the likelihood-ratio values obtained for each possible identity claim. Decisions are made independently for each subregion at the same level, and only the most significant one is kept. The following sections will further detail the process of model training and recognition score computation for a single generic subregion, while also motivating the proposed UBM framework.

### 3.1. Universal Background Model

The universal background modeling strategy was initially proposed in the field of voice biometrics [29]. Its framework can be easily understood if the problem of biometric verification is interpreted as a basic hypothesis test. Given a biometric sample *Y* and a claimed ID, *S*, we define:

$$\begin{array}{c} H_{0}=Y\;\text{belongs to}\;S\\ H_{1}=Y\;\text{does not belongs to}\;S \end{array}$$

as the null and alternative hypothesis, respectively. The optimal decision is made by a likelihood-ratio test:

(1) $$\frac{p\left(Y|H_{0}\right)}{p\left(Y|H_{1}\right)}\{\begin{array}{cc} \geq \theta & \text{accept}\;H_{0}\\ <\theta & \text{accept}\;H_{1} \end{array}$$

where *θ* is the decision threshold for accepting or rejecting *H*_0_ and *p*(*Y* ∣ *H_i_*) is the likelihood of observing *Y* knowing that *H_i_* is true. The goal of a biometric verification system can, thus, be accomplished by the computation of the likelihood values *p*(*Y*∣*H*_0_) and *p*(*Y*∣*H*_1_) for a given sample. It is intuitive to note that *H*_0_ will correspond to a model λ*_hyp_* that characterizes the hypothesized individual, whereas *H*_1_ will represent the alternative hypothesis, that is the model of all of the alternatives to the hypothesized individual, $\lambda_{\bar{\text{hyp}}}$.

The computation of *p*(*Y*∣*H_i_*) depends on the specific strategy chosen for data modeling. If *H*_0_ and *H*_1_ correspond to a pair of generative models, trained on sets of genuine and impostor data, respectively, then the *p*(*Y*∣*H_i_*) values can be roughly expressed as the projections of biometric sample *Y* onto each of these models. This formulation motivates the need for a model that successfully covers the space of alternatives to the hypothesized identity. The most common designation in the literature for such a model is the universal background model or UBM [30]. Such a model must be trained on a rather large set of data, so as to faithfully cover a representative user space.

Even though the UBM approach was initially proposed for verification mode, we extrapolate its rationale for identification systems. Instead of performing a single one *vs.* one likelihood-ratio test and checking the validity of the condition presented in Equation (1), a one *vs.* all approach may alternatively be considered. Given an unknown sample, the most likely identity, *Id_max_*, will correspond to the highest likelihood-ratio value, amongst all enrolled users:

(2) $$Id_{\max}=\underset{i}{\mathrm{argmax}}\;\left(\frac{p\left(Y|H_{0}^{\left(i\right)}\right)}{p\left(Y|H_{1}\right)}\right)$$

where $H_{0}^{\left(i\right)}$ represents the model describing user *i*.

Defining an objective way of quantifying *p*(*Y*∣*H*_0_) and *p*(*Y*∣*H*_1_) becomes, thus, the true challenge when following this approach. In the following sections, we analyze in detail the strategies chosen to model both λ*_hyp_* and $\lambda_{\bar{\text{hyp}}}$.

### 3.2. Hypothesis Modeling

In the present work, we chose Gaussian mixture models (GMM) to model both the UBM, *i.e.*, $\lambda_{\bar{\text{hyp}}}$, and the individual-specific models (IDSM), *i.e.*, λ*_hyp_*. Such models are capable of capturing the empirical probability density function of a given set of feature vectors, so as to faithfully model their intrinsic statistical properties [28]. The choice of GMM to model feature distributions in biometric data is extensively motivated in many works of related areas. From the most common interpretations, GMMs are seen as capable of representing broad “hidden” classes, reflective of the unique structural arrangements observed in the analyzed biometric traits [28]. Besides this assumption, Gaussian mixtures display both the robustness of parametric unimodal Gaussian density estimates, as well as the ability of non-parametric models to fit non-Gaussian data [31]. This duality, alongside the fact that GMM have the noteworthy strength of generating smooth parametric densities, confers such models a strong advantage as generative models of choice. For computational efficiency, GMM models are often trained using diagonal covariance matrices. This approximation is often found in the biometrics literature, with no significant accuracy loss associated [32].

All models are trained on densely-sampled sets of scale-invariant feature transform (dSIFT) keypoint descriptors, extracted from previously normalized facial sub-regions. Illumination normalization is performed using the Weber-face approach [33]. Even though traditional SIFT descriptors present invariance to a set of common undesirable factors (image scaling, translation, rotation), thus conferring them a strong appeal in unconstrained biometrics, the fact that they fail to adapt to heterogeneous illumination conditions severely hinders their practical use in real-life applications. The normalization step is, therefore, of the utmost importance.

Dense SIFT is a variation of the traditional SIFT methodology [34], where keypoint descriptors are extracted in a roughly equivalent manner to running SIFT on a dense grid of locations at a fixed scale and orientation [35]. Dense sampling mitigates the potential errors introduced by the detection of an unreliable set of interest points in its sparse counterpart [36]. In the present work, we train GMMs on the set of all densely-sampled keypoint descriptors from all individuals (UBM) and adapt individual models using data from specific subjects alone (IDSM), achieving a stable summary of the image content for every enrolled user.

Originally, SIFT descriptors were defined in 128 dimensions. However, we chose to perform a PCA, as suggested in [37], reducing the dimensionality to 32. Such a reduction allows not only a significant reduction in the computational complexity of the training phase, but also an improved distinctiveness and robustness to the extracted feature vectors, especially as far as image deformation is concerned [38]. We computed the principle components from the same set of keypoint descriptors used to train the UBM.

### 3.2.1. UBM Parameter Estimation

To train the universal background model, a large amount of “impostor” data, *i.e.*, a set composed of data from all the enrolled individuals, is used, so as to cover a wide range of possibilities in the individual search space [37]. The training process of the UBM is simply performed by fitting a *k*-mixture GMM to the set of PCA-reduced feature vectors extracted from all of the “impostors”.

If we interpret the UBM as an “impostor” model, its “genuine” counterpart can be obtained by adaptation of the UBM's parameters, $\lambda_{\bar{\text{hyp}}}$ using individual specific data. For each enrolled user, *n*, an IDSM, defined by parameters λ*_hyp_n__*, is therefore obtained. The adaptation process will be outlined in the following section.

#### 3.2.2. MAP Adaptation

IDSMs are generated by the tuning of the UBM parameters, in a maximum *a posteriori* (MAP) sense, using individual-specific biometric data. This approach provides a tight coupling between the IDSM and the UBM, resulting in better performance and faster scoring than uncoupled methods, as well as a robust and precise parameter estimation, even when only a small amount of data is available [37]. The adaptation process consists of two main estimation steps. First, for each component of the UBM, a set of sufficient statistics is computed from a set of *M* individual-specific feature vectors, *X* = {**x**_1_, …, **x***_M_*}:

(3) $$n_{i}=\sum_{m=1}^{M}p\left(i|x_{m}\right)$$

(4) $$E_{i}\left(x\right)=\frac{1}{n_{i}}\sum_{m=1}^{M}p\left(i|x_{m}\right)x_{m}$$

(5) $$E_{i}\left({xx}^{t}\right)=\frac{1}{n_{i}}\sum_{m=1}^{M}p\left(i|x_{m}\right)x_{m}x_{m}^{t}$$

where *p*(*i*∣**x***_m_*) represents the probabilistic alignment of **x***_m_* into each UBM component. Each UBM component is then adapted using the newly-computed sufficient statistics and considering diagonal covariance matrices. The update process can be formally expressed as:

(6) $${\hat{w}}_{i}=\left[\alpha_{i}n_{i}/M+\left(1-\alpha_{i}\right)w_{i}\right]\xi$$

(7) $${\hat{\mu }}_{i}=\alpha_{i}E_{i}\left(x\right)+\left(1-\alpha_{i}\right)\mu_{i}$$

(8) $${\hat{\sum }}_{i}=\alpha_{i}E_{i}\left({\text{xx}}^{t}\right)+\left(1-\alpha_{i}\right)\left(\sigma_{i}\sigma_{i}^{t}+\mu_{i}\mu_{i}^{t}\right)-{\hat{\mu }}_{i}{\hat{\mu }}_{i}^{t}$$

(9) $$\sigma_{i}=\text{diag}\left(\sum_{i}\right)$$

where {*w_i_*, ***μ****_i_*, ***σ****_i_*} are the original UBM parameters and {*ŵ_i_*, ***μ̂****_i_*, ***σ̂****_i_*} represent their adaptation to a specific speaker. To assure that Σ*_i_ w_i_* = 1, a weighting parameter *ξ* is introduced. The *α* parameter is a data-dependent adaptation coefficient. Formally, it can be defined as:

(10) $$\alpha_{i}=\frac{n_{i}}{r+n_{i}}$$

The relevance factor *r* weights the relative importance of the original values and the new sufficient statistics. In the present, work we set *r* = 16.

### 3.3. Hierarchical Decision

The whole training process is repeated at most *M* times for each of *M* facial subregions, defined *a priori*. In the present work, we used the *M* = 14 regions previously exemplified in Figure 1.

Traditionally, the recognition phase with new data from an unknown source is a fairly simple process. The new test data, *X_test_* = {**x**_*t*,1_,…, **x**_*t*,*N*_}, where **x**_*t*,*i*_ is the *i*-th PCA-reduced SIFT vector extracted from a given subregion *m* of test subject *t*, is projected onto both the UBM and either the claimed IDSM (in verification mode) or all such models (in identification mode). The recognition score, *s*_*t*,*m*_, is obtained as the average likelihood-ratio of all keypoint descriptors $x_{t,i,}s_{t,m}=\frac{1}{N}\sum_{i=1}^{N}s_{t,m}^{\left(i\right)}$. The decision is then carried out by checking the condition presented in Equation (1), in the case of verification, or by detecting the maximum likelihood-ratio value for all enrolled IDs (Equation (2)), in the case of identification.

Such a decision step represents the second big advantage of the UBM approach. The ratio between the IDSM and the UBM probabilities of the observed data is a more robust decision criterion than relying solely on the IDSM probability. This results from the fact that some subjects are more prone to generate high likelihood values than others, *i.e.*, some people have a more “generic” look than others. The use of a likelihood ratio with a universal reference works as a normalization step, mapping the likelihood values in accordance with their global projection. Without such a step, finding a global optimal value for the decision threshold, *θ*, presented in Equation (1), would be a far more complex process.

In an attempt to integrate meaningful information from the *M* facial subregions and to deal better with partial or missing data situations originated by occlusions, an hierarchical recognition framework is proposed. The rationale behind the methodology described below can be easily understood if some studies of the human brain's cognitive mechanisms of perception are taken into consideration. One such work, proposed in 1977 by David Navon [12], describes the aforementioned mechanism as a hierarchical process, where holistic representations precede more detailed local features. If we interpret this perception mechanism in the scope of human face recognition, we can conclude that an attempt to classify the face at a global level is the starting point to the whole recognition process, whereas increasingly detailed descriptions are only taken subsequently if necessary. This conceptual description of the human perception mechanism serves as the basis for the proposed hierarchical recognition algorithm, whose flowchart is presented in Figure 1c.

The main steps of the proposed hierarchical identification algorithm are as described below:

(1) Initialization: Starting with the full-face image from an unknown user, *I*_0_, a densely-sampled grid of SIFT keypoint descriptors is extracted; the likelihood values, $l_{t,0}^{{\text{IDSM}}_{t}}$ and $l_{t,0}^{{\text{UBM}}_{0}}$, are then computed for the IDSM of every enrolled user *t* ∈ {1..*T*} and the UBM of the tested region; the recognition scores for every possible identity, ***s****_t_*_,0_ = {*s*_1,0_, …, *s_T_*_,0_}, are then computed through Equation (1).
(2) Certainty index computation: The certainty index of a given region, *c_m_*, measures how likely it is that the obtained vector of likelihood ratios, ***s***_*t*,*m*_, corresponds with the ideal case of a single correct identity match. If no false positives corrupt the vector of likelihood ratios obtained for a single image, a significant difference will be observed between the highest value, ***s***_t*,*m*_, (true identity) and the average of all other values, $\frac{1}{T-1}\sum_{t=1,t\neq t*}^{T}{\text{s}}_{\text{t},\text{m}}$, (average impostor). The certainty index can thus be interpreted as a degree of separability between these two quantities:
  (11) $$c_{m}={\text{s}}_{\text{t}*,\text{m}}-\frac{1}{T-1}\sum_{t=1,t\neq t*}^{T}{\text{s}}_{\text{t},\text{m}}$$
(3) Decision to go to next level: If the *c_m_* value exceeds a previously optimized threshold, *θ_l_*, the maximum likelihood-ratio decision is accepted. When *c_m_* < *θ_l_*, however, the algorithm will consider that an analysis at a more detailed level is necessary to achieve a decision with a higher degree of confidence. At this point, the algorithm proceeds to the next level, working on subregions *I*_1–2_, the second in the hierarchical chain depicted in Figure 1. When one level is composed by multiple subregions, each one of them is treated independently, and only the maximum *c_m_* value among them is considered for the decision criterion:
  (13) $$c_{m}=\{\begin{array}{cc} c_{0} & \text{if}l=1\\ \max\left\{c_{1},c_{2}\right\} & \text{if}l=2\\ \max\left\{c_{3},c_{4}\right\} & \text{if}l=3\\ \max\left\{c_{5},c_{6},c_{7}\right\} & \text{if}l=4\\ \max\left\{c_{8},c_{9},c_{10}\right\} & \text{if}l=5\\ \max\left\{c_{11},c_{12},c_{13},c_{14}\right\} & \text{if}l=6 \end{array}$$
(4) Repeat: Steps 1 to 3 are hierarchically repeated for every level until *c_m_* > *θ_l_*. If all *L* levels are considered and none is able to achieve a significant decision, the decision corresponding to the highest value of *c_m_*, $\underset{m}{\max}(c_{m})$, amongst all *L* levels is considered.

As the proposed algorithm is capable of performing recognition without the need of processing all subregions, computational speed is significantly improved over simpler approaches that explore the fusion of all local recognition scores. Furthermore, the proposed algorithm is capable of automatically deciding if a more detailed exploration of local features is necessary or if the information obtained up to a certain point is enough to make a decision. This autonomy alongside the intuitive notion of the global precedence hypothesis are the most notorious strengths of the proposed recognition algorithm.

An alternative to deciding non-classified images at the end of the hierarchical chain was also considered. In this new setting, if no level is capable of making a decision according to the aforementioned criteria, images are kept as “doubtful”, and no decision is made, in a process similar to classification with a reject option [39]. This approach may be thought of as a viable alternative for real-life applications, where feedback to the user can be explored to adjust the environmental conditions in severely unconstrained scenarios.

## 4. Results and Discussion

### 4.1. Experimental Setups

The proposed algorithm was tested on the Database of Faces (formerly “The ORL Database of Faces”), the Extended Yale Face Database B and the AR face database. All tested databases are widely known for their diversity of pose, illumination and occlusion conditions, respectively. The next sections outline the main features of each database, as well as the experimental setup chosen for training and testing with each of them in the scope of the present work.

#### 4.1.1. ORL

**Figure 2.**
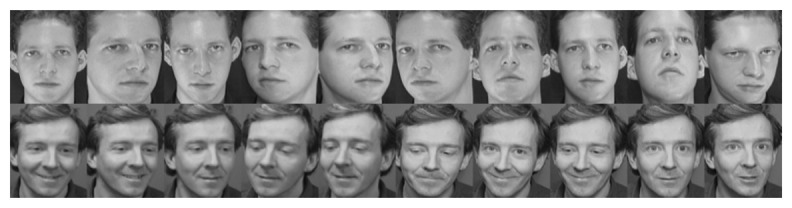
Example images from two subjects of the ORLdatabase.

The Database of Faces (formerly “The ORL Database of Faces”) [40] contains 400 images from 40 subjects, divided equitably with a total of 10 images per individual. Images were taken at different points in time with variable lighting, expression and pose conditions. For performance assessment, we use a single sample from each individual for training and the remaining nine images for testing. This process is repeated for each possible training image, and the performance is computed as the average of the 10 runs. An alternative approach is also explored by using multiple templates per subject instead. In this new setup, the first five samples per subject are used for training, while the remaining five samples are used for testing. Example images from two subjects may be observed in Figure 2.

#### 4.1.2. Extended Yale B

**Figure 3.**
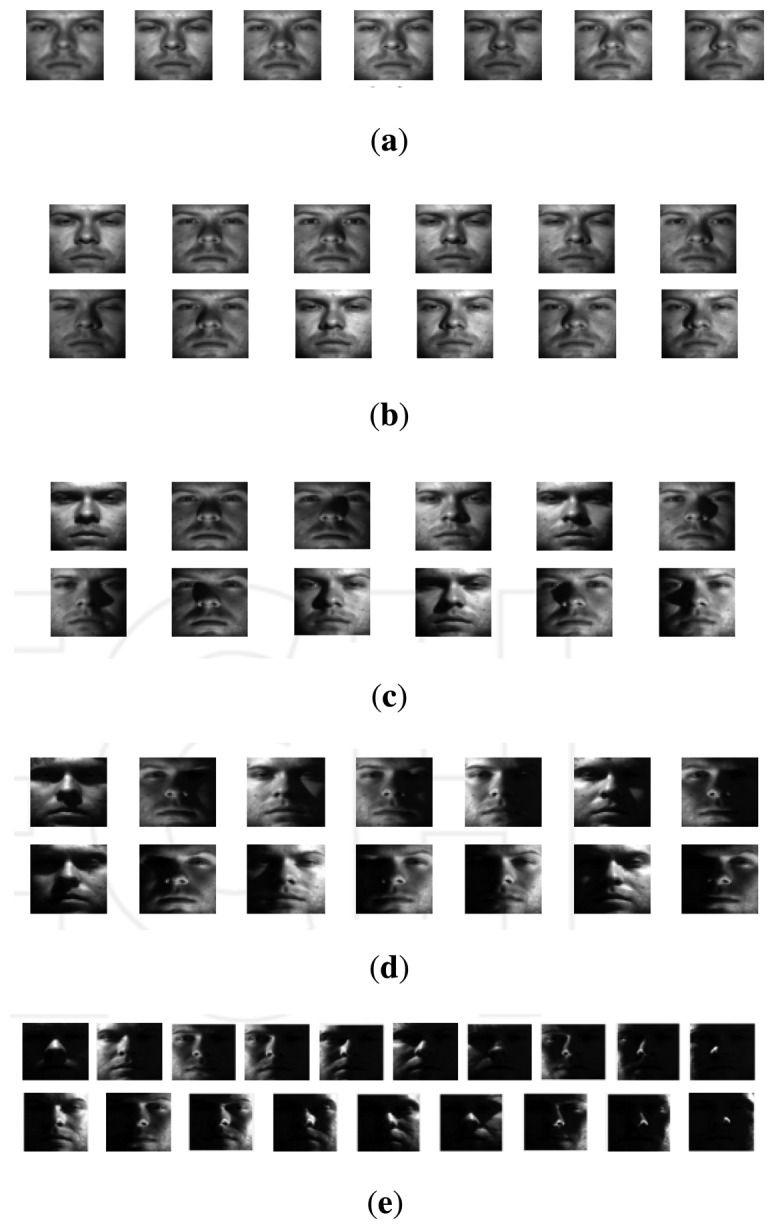
All images from a single subject enrolled in the Extended Yale B database. Images (**a**) to (**e**) correspond to Subsets 1 to 5, respectively.

The Extended Yale Face Database B [41] is composed of 2432 images corresponding to a total of 38 individuals. All images are frontal faces acquired under varying illumination conditions. The database is divided into five subsets, numbered 1 to 5, according to the ranges of angles between the light source direction and the camera axis. An example of all images from a single subject may be observed in Figure 3.

All images from Subset 1 were used for model training, while all other subsets were tested independently, so as to better assess the robustness of the proposed algorithm to a variety of illumination conditions.

#### 4.1.3. AR

**Figure 4.**
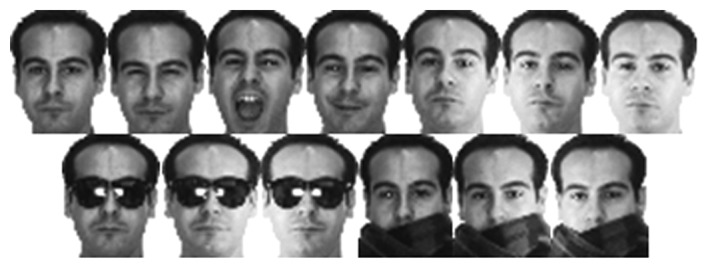
Example images from one subject of the ARdatabase.

The AR database [42] contains over 4000 frontal face images from 126 individuals, acquired under variable illumination, expression and occlusion. Occlusions can be divided into two main categories: sunglasses and scarf. An example of all images from a single individual is presented in Figure 4.

All unoccluded images from every individual are chosen to train both the local UBMs and IDSMs. The remaining scarf and sunglasses occluded images are tested separately, so as to better analyze the consistency of the proposed algorithm when exposed to variable types of occlusion.

#### 4.1.4. Performance Analysis

**Figure 5.**
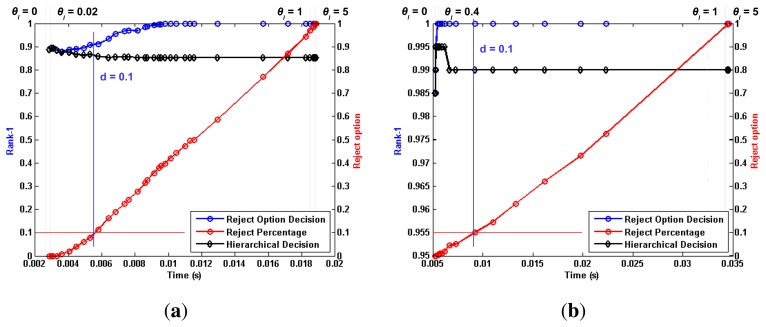
Main results obtained with the proposed methodology for the sunglasses and scarf images of the ORL face database. Each plotted point represent a specific value of parameter *θ_l_*, ranging from [0, ∞].

**Figure 6.**
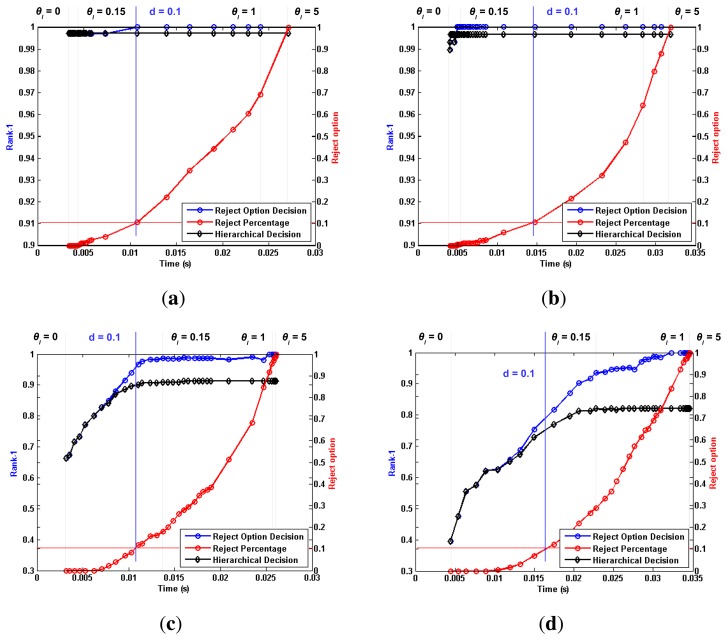
Main results obtained with the proposed methodology for Subsets 2–5 of the Extended Yale B Face database.

**Figure 7.**
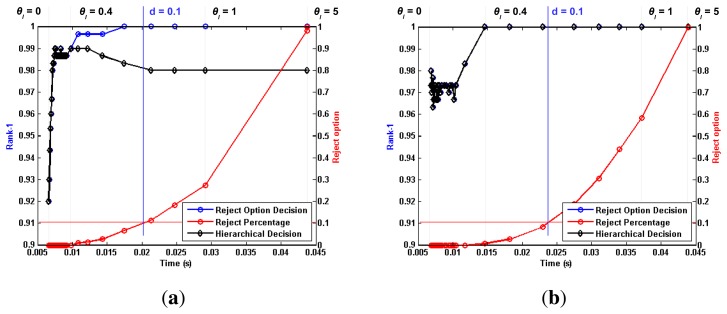
Main results obtained with the proposed methodology for the sunglasses and scarf images of the AR face database. Each plotted point represent a specific value of parameter θ*_l_*, ranging from [0, ∞].

Figures 5, 6 and 7 depict the most relevant results obtained by the proposed algorithm for the ORL, Extended Yale B and AR databases, using the previously mentioned training and testing setups. Furthermore, Tables 1, 2 and 3 present a comparison between the proposed work and the reported performance in some recent works, performed under similar experimental conditions. We chose to assess the rate of correctly identified individuals, by checking if the true identity is present among the *N* highest ranked identities. The *N* parameter is generally referred to as rank. This allows us to define the Rank-1 recognition rate, *r*_1_, as the recognition rate at *N* = 1.

Each plotted point refers to a single *θ_l_* value, ranging from [0, ∞]. For each tested *θ_l_* value, we plotted a series of performance metrics against the corresponding average processing time per image. Using the *θ_l_* = 0.1 vertical line from Figure 7a as a reference, we can distinguish three metrics that are common to all tested setups. The black line represents the evolution of *r*_1_ when “doubtful” images are classified through the aforementioned criterion, $\underset{m}{\max}(c_{m})$. When no such *a posteriori* classification is performed, a set of images is left unclassified, as no level from the hierarchical chain was shown to present enough detail to perform an accurate recognition. The red line represents the evolution of the “doubtful” image percentage with increasing *θ_l_* values. It is intuitive to note that lower *θ_l_* values generate lower reject-option classification ratios, as a smaller dissimilarity between individuals will still trigger a “certain” decision. If no “doubtful” images are considered in the performance evaluation of the proposed algorithm, then the *r*_1_ computation is performed only with respect to the non-rejected images. The blue line represents this alternative evaluation, and the corresponding reject percentage can be easily traced to the equivalent *θ_l_* point in the red line. Taking, once again, the *θ_l_* = 0.1 vertical line from Figure 7a as a reference, we can see that the proposed methodology yields a *r*_1_ value of roughly 98.00%. If the approximately 30% “doubtful” images are not considered for evaluation, the *r*_1_ value increases to 100.00%, with respect to the non-rejected images.

For a better comprehension and deeper analysis of the method, we chose to assess performance in three specific points:

(1) Optimal ***θ****_l_* value: We consider the optimal *θ_l_* value as the point where a visible performance plateau is achieved in the time *vs.* performance plot. For the *AR* database, this value was set to *θ_l_* = 0.4, whereas for the Extended Yale B Face database, it was set to *θ_l_* = 0.15. For the ORL database, the *θ_l_* value was optimized for each of the aforementioned experimental setups. For the single template approach, the value was set to *θ_l_* = 0.02, while for multiple templates, it was set to *θ_l_* = 0.15.
(2) ***θ****_l_* → ∞: extreme behavior when the *θ_l_* parameter is set to high values.
(3) ***d*** < 0.1 : point where the ratio of non-classified images at the end of the hierarchical chain reaches 10% of all tested images.

A thorough analysis of Figures 5, 6 and 7 leads to some interesting conclusions regarding the behavior of the proposed algorithm under variable image acquisition conditions. The performance metric *r*_1_ was computed for a set of *θ_l_* values, ranging from [0, ∞]. It is trivial to understand that lower values of *θ_l_* will lead the proposed algorithm to an extreme case where all images are classified in the first level of information, *i.e.*, the full face, thus reducing the computational complexity and average processing time. On the other hand, when *θ* → ∞, all images will reach the end of the hierarchical chain without a certain decision having been made. In this opposite extreme behavior, all images are classified *a posteriori* using information from every level, with a significant increase in both computational complexity and performance. With such a wide variety of possibilities, we chose to analyze the global behavior of the proposed work by plotting the evolution of *r*_1_ values against the average processing time, when the *θ_l_* parameter goes from [0, ∞].

Regarding the evolution of *r*_1_ for variable *θ_l_* values, we might point out the considerable drop in performance under less ideal conditions, when *θ* → 0. Whereas Subsets 2 and 3 from the Extended Yale B database show excellent performance from both aforementioned approaches, rivaling those obtained for *θ* → ∞, such behavior is highly compromised for the more challenging scenarios of Subsets 4 and 5. In such cases, the observed drop in performance is less significant for higher *θ_l_* values. Furthermore, the results obtained for the AR database stress that the proposed methodology is capable of consistently presenting high performance regardless of the acquisition conditions and noise factors. Whereas the Extended Yale B database presented the challenge of heterogeneous illumination, which might be understood as a “natural” source of occlusion, the AR database presents the challenge of spatially coherent occlusion regions. For both cases, performance observed for the proposed hierarchical methodology rivals the state-of-the-art. Regarding the *θ* → ∞ case, the expectation that the best results would be consistently observed for this extreme scenario was fulfilled, at the expense of higher computation complexity and average processing time. Nevertheless, if no time constraints exist in the application scenario in which the proposed methodology is implemented, higher values of *θ_l_* seem the ideal choice. The ORL database maintains a somewhat stable performance regardless of the chosen value for the *θ_l_* parameter. As the proposed hierarchical methodology is contextually motivated for occlusion scenarios and no such conditions are present in this database, the observed behavior fits the expectations. Regardless of the absence of occlusions in the ORL images, the reject option classifier still proves a useful tool at discriminating “doubtful” images and increasing the reliability of the decisions obtained for the “non-doubtful” ones.

Tables 1, 2 and 3 present a comparative analysis between the proposed methodologies and some state-of-the-art works, in similar experimental setups, for the AR and Extended Yale B databases, respectively. It is readily observed that the proposed algorithm presents the most consistent and robust behavior, regardless of the nature of the present occlusions, for the AR face database. While the work by Li *et al.* [6] presents higher performance for sunglasses occlusion, their performance regarding the alternative scarf occlusion is considerably lower than the one obtained with the proposed algorithm. Min *et al.* [5] present a work that suffers from exactly the opposite problem, with good performance observed for scarf occlusion, but lower results for sunglasses. The aforementioned works seem to suffer from overfitting to certain classes of images and lack the robustness to adapt to new cases. Such robustness to the nature and location of the occlusion can be observed for both the proposed methodology, as well as the works from Morelli *et al.* [46] and Qian *et al.* [23]. Both of these works show a similar trend to the proposed algorithm and high performance for the whole database. Alongside our work, and to the best of our knowledge, they represent the state-of-the-art performance for the AR database in unconstrained face recognition. A similar analysis can be made for both setups under which experiments were carried out with the ORL database. The best performance obtained with the proposed algorithm is on par with the best value found in the literature, in the work by Geng and Jiang [19]. This observation leads to the conclusion that the proposed methodology is capable of achieving state-of-the-art performance for a wider variety of scenarios besides occlusion. The aforementioned observation that performance seems to be independent of the chosen value for the *θ_l_* parameter may, however, indicate that a simpler approach than the whole hierarchical chain might also output good results. Using simply the first level, *i.e.*, the full-face images, the *r*_1_ values observed for the single and multiple template setups were 88.10% ± 1.05% and 98.5%, respectively. These results differ very little from the optimal results presented in Table 1, thus corroborating the expectation of good performance for a less computationally complex approach.

Regarding the Extended Yale B Face database, our algorithm is shown to perform similar to state-of-the-art performance for all subsets, except Subset 5. Even for this subset, the obtained performance is still higher than a few recently published works, even though no direct comparison can be performed with the work by Cho *et al.* [11], whose authors claim that its images are “excluded from the experiment because the illumination conditions are so severe that some images are difficult to recognize even to the naked eye”. Observing the behavior of the blue and red lines for high threshold values, we note that a significant amount of images in this subset exceeds the recognition capability of the proposed methodology. The fact that the blue and black lines diverge greatly, when compared to the results observed in other subsets, shows that posterior classification by the maximum *c_m_* value may not be the most beneficial approach when working with such severely degraded images. In such cases, the reject option classification yields considerably higher performance than the posterior classification of doubtful cases, even for low rejection ratios. It is easily noted how the blue and black lines start to diverge for a value of *d* ≪ 0.1, as a result of this behavior.

### 4.2. Implementation Details

The proposed algorithm was developed in MATLAB R2012a and tested on a PC with 3.40 GHz Intel(R) Core(TM) i7-2600 processor and 8 GB RAM. To train the GMMs, we used the Netlab toolbox [50], whereas dSIFT keypoint description was performed using the VLFeat toolbox [51].

## 5. Conclusions and Future Work

In the present work, we propose an algorithm for face recognition under unconstrained settings, such as heterogeneous illumination and severe occlusions. By training models that only describe features of limited regions of the face, we confer our algorithm a robustness to events where all other regions are occluded. In an attempt to replicate the global precedent hypothesis of the human brain's cognitive mechanisms of perception, we designed an innovative hierarchical recognition algorithm, where face recognition is performed locally only if a more global representation is not capable of achieving a decision with a high degree of certainty. Even though good performance was observed for a wide variety of non-ideal conditions, some ideas are worth noting for future research in the area.

First of all, the choice of SIFT keypoint descriptors distributed in a dense grid is based on a series of assumptions, like constant scale and orientation, that might not always hold. Besides these assumptions, we performed no comparative analysis with other descriptors that might prove more appropriate for face description in less controlled acquisition scenarios. The feature extraction algorithm proposed by Miao and Jiang [52], for example, is reported to be more efficient at extracting interest points from human face images than the SIFT approach followed in the present work and might prove to be an interesting alternative. Still, regarding face representation, no alternative pre-processing methods were considered besides the aforementioned Weber-faces approach. Given that the most significant performance drop of the proposed algorithm was observed for images acquired under significantly low illumination, alternative normalization techniques might deserve further research. A different approach might be considered, as previously mentioned, by introducing a feedback mechanism to the recognition system, with the user being notified when the reject option is triggered in the end of the hierarchical chain. This could allow a user to actively adapt the environmental conditions so as to facilitate a more certain decision.

The choice of the fittest *θ_l_* value for each specific application might also motivate a more detailed study. The hypothesis that some users are easier to identify than others inside a given population, an effect known as the Doddington zoo effect [53], suggests that parameter optimization in biometric applications might benefit from a more individual-specific approach. Besides this observation, we fixed the *θ_l_* value for all levels of the hierarchical chain. We assumed that due to the normalization of the likelihood-ratio provided by the UBM, recognition scores at different levels share a similar range of values. Even though this assumption seems valid enough in the proposed framework, analyzing the effect of level-specific threshold values might bring about slight increases in performance.

In a final consideration, the proposed work was carried out fully on frontal face images. Most real-life applications in unconstrained scenarios should not enforce such a rigid constraint, and thus, the proposed algorithm should be capable of coping with pose variations. Two alternatives to approach this problem could be the training of multiple models describing individual poses or the training of a single model built on images acquired under multiple poses. Further research into both alternatives would be needed before either of them can be considered as the most fit to expand the proposed framework.

## Figures and Tables

**Table 1.** Comparison between the obtained *r*_1_ values for the ORL database and some state-of-the-art algorithms.

**Work**	**Single Template *μ* ± *σ***	**Multiple Templates**
Proposed*_θ_*_=0.02∣0.15_	**89.45**% ± **1.24**%	**99.5**%
Proposed*_θ_*_→∞_	85.79% ± 1.85%	99.00%
Proposed*_d_*_<0.1_	***93.60***% ± ***1.85***%	***100.0***%

Geng and Jiang [19]	89.0%	**99.5**%
Zhang *et al.* [43]	–	85.0%
Xiao *et al.* [44]	–	97.50
Xu *et al.* [45]	–	97.10

**Table 2.** Comparison between the obtained *r*_1_ values for the AR database and some state-of-the-art algorithms.

**Work**	**AR Sunglasses**	**AR Scarf**	**AR Average**
Proposed*_θ_*_=0.4_	**100.0**%	99.00%	**99.50**%
Proposed*_θ_*_→∞_	**100.0**%	98.00%	99.00%
Proposed*_d_*_<0.1_	***100.0*%**	***100.0*%**	***100.0*%**

Min *et al.* [5]	75.00%	92.08%	83.54%
Li *et al.* [6]	97.00%	52.00%	74.50%
Morelli Andrés *et al.* [46]	97.46%	99.15%	98.31%
Qian *et al.* [23]	99.00%	**100.0**%	**99.50**%

**Table 3.** Comparison between the obtained *r*_1_ values for the Extended Yale B database and some state-of-the-art algorithms.

**Work**	**Yale B *SS*_2_**	**Yale B *SS*_3_**	**Yale B *SS*_4_**	**Yale B *SS*_5_**
Proposed*_θ_*_=0.15_	99.71%	99.66%	90.91%	81.99%
Proposed*_θ_*_→∞_	99.71%	99.66%	91.20%	81.99%
Proposed*_d_*_<0.1_	***100.0*%**	***100.0*%**	*96.68*%	*81.66*%

Cho *et al.* [11]	**100**%	100%	99.2%	—
Yang *et al.* [47]	—	—	90.2%	47.9%
Jiang *et al.* [48]	**100**%	99.12%	83.46%	57.84%
Jia *et al.* [49]	**100**%	**100**%	**97.15**%	**96.13**%

## References

[1] Prabhakar S., Pankanti S., Jain A.K. (2003). Biometric recognition: Security and privacy concerns. IEEE Secur. Priv..

[2] Jain A., Hong L., Pankanti S. (2000). Biometric identification. Commun. ACM.

[3] Monteiro J.C., Sequeira A.F., Oliveira H.P., Cardoso J.S. (2014). Robust iris localisation in challenging scenarios. Commun. Comput. Inform. Sci..

[4] Castrillón M., Déniz O., Hernández D., Lorenzo J. (2011). A comparison of face and facial feature detectors based on the Viola–Jones general object detection framework. Mach. Vis. Appl..

[5] Min R., Hadid A., Dugelay J.L. (2014). Efficient detection of occlusion prior to robust face recognition. Sci. World J..

[6] Li Y., Meng L., Feng J., Wu J. (2014). Downsampling sparse representation and discriminant information aided occluded face recognition. Sci. China Inf. Sci..

[7] Karande K.J., Talbar S.N. (2014). Independent Component Analysis of Edge Information for Face Recognition.

[8] Tanaka J.W., Farah M.J. (1993). Parts and wholes in face recognition. Q. J. Exp. Psychol..

[9] Schwaninger A., Schumacher S., Bülthoff H., Wallraven C. Using 3D computer graphics for perception: The role of local and global information in face processing.

[10] Gold J.M., Mundy P.J., Tjan B.S. (2012). The perception of a face is no more than the sum of its parts. Psychol. Sci..

[11] Cho H., Roberts R., Jung B., Choi O., Moon S. (2014). An Efficient Hybrid Face Recognition Algorithm Using PCA and GABOR Wavelets. Int. J. Adv. Robot. Syst..

[12] Navon D. (1977). Forest before trees: The precedence of global features in visual perception. Cogn. Psychol..

[13] Turk M., Pentland A. (1991). Eigenfaces for recognition. J. Cogn. Neurosci..

[14] Belhumeur P.N., Hespanha J.P., Kriegman D. (1997). Eigenfaces vs. fisherfaces: Recognition using class specific linear projection. IEEE Trans. Pattern Anal. Mach. Intell..

[15] Cootes T.F., Edwards G.J., Taylor C.J. (1998). Active appearance models. Lect. Notes Comput. Sci..

[16] Liao S., Jain A.K. Partial face recognition: An alignment free approach.

[17] Nallammal N., Radha V. (2013). Comparative Analysis of Partial Occlusion Using Face Recognition Techniques. Int. J. Image Proc. (IJIP).

[18] Oh B.S., Toh K.A., Choi K., Beng Jin Teoh A., Kim J. (2012). Extraction and fusion of partial face features for cancelable identity verification. Pattern Recognit..

[19] Geng C., Jiang X. (2011). Face recognition based on the multi-scale local image structures. Pattern Recognit..

[20] Taigman Y., Yang M., Ranzato M., Wolf L. Deepface: Closing the gap to human-level performance in face verification.

[21] Wright J., Yang A.Y., Ganesh A., Sastry S.S., Ma Y. (2009). Robust face recognition via sparse representation. IEEE Trans. Pattern Anal. Mach. Intell..

[22] Zhou Z., Wagner A., Mobahi H., Wright J., Ma Y. Face recognition with contiguous occlusion using markov random fields.

[23] Qian J., Yang J., Zhang F., Lin Z. Robust Low-Rank Regularized Regression for Face Recognition with Occlusion.

[24] Wang J., Lu C., Wang M., Li P., Yan S., Hu X. (2014). Robust Face Recognition via Adaptive Sparse Representation. IEEE Trans. Cybern..

[25] Shen Y., Hu W., Yang M., Wei B., Lucey S., Chou C.T. Face recognition on smartphones via optimised sparse representation classification.

[26] Jian Z., Luxi H., Jian J., Yu X. (2014). A Fast Iterative Pursuit Algorithm in Robust Face Recognition Based on Sparse Representation. Math. Probl. Eng..

[27] Zhang W., Shan S., Chen X., Gao W. (2007). Local Gabor binary patterns based on Kullback–Leibler divergence for partially occluded face recognition. IEEE Signal Process. Lett..

[28] Reynolds D., Quatieri T., Dunn R. (2000). Speaker verification using adapted Gaussian mixture models. Digit. Signal Process..

[29] Povey D., Chu S.M., Varadarajan B. Universal background model based speech recognition.

[30] Reynolds D.A. An overview of automatic speaker recognition technology.

[31] Reynolds D. (2008). Gaussian mixture models. Encyclopedia of Biometric Recognition.

[32] Xiong Z., Zheng T., Song Z., Soong F., Wu W. (2006). A tree-based kernel selection approach to efficient Gaussian mixture model–universal background model based speaker identification. Speech Commun..

[33] Wang B., Li W., Yang W., Liao Q. (2011). Illumination normalization based on weber's law with application to face recognition. IEEE Signal Process. Lett..

[34] Lowe D.G. (2004). Distinctive image features from scale-invariant keypoints. Int. J. Comput. Vis..

[35] Bosch A., Zisserman A., Muñoz X. (2006). Scene classification via pLSA. Lect. Notes Comput. Sci..

[36] Simonyan K., Parkhi O.M., Vedaldi A., Zisserman A. Fisher vector faces in the wild.

[37] Shinoda K., Inoue N. (2013). Reusing Speech Techniques for Video Semantic Indexing [Applications Corner]. IEEE Signal Process. Mag..

[38] Ke Y., Sukthankar R. PCA-SIFT: A more distinctive representation for local image descriptors.

[39] Herbei R., Wegkamp M.H. (2006). Classification with reject option. Can. J. Stat..

[40] Samaria F.S., Harter A.C. Parameterisation of a stochastic model for human face identification.

[41] Georghiades A., Belhumeur P., Kriegman D. (2001). From Few to Many: Illumination Cone Models for Face Recognition under Variable Lighting and Pose. IEEE Trans. Pattern Anal. Mach. Intell..

[42] Martinez A.M. (1998). The AR Face Database.

[43] Zhang D.L., Qiao J., Li J.B., Qiao L.Y., Chu S.C., Roddick J.F. (2014). Optimizing Matrix Mapping with Data Dependent Kernel for Image Classification. J. Inf. Hiding Multimed. Signal Process..

[44] Xia S., Zheng G., Ma Y., Ma X. A. Novel Super-resolution Approach Based on Supervised Canonical Correlation Analysis.

[45] Xu W., Lee E.J. (2014). Face Recognition Using Wavelets Transform and 2D PCA by SVM Classifier. Int. J. Multimed. Ubiquitous Eng..

[46] Morelli Andrés A., Padovani S., Tepper M., Jacobo-Berlles J. (2014). Face recognition on partially occluded images using compressed sensing. Pattern Recognit. Lett..

[47] Yang J., Qian J., Luo L., Zhang F., Gao Y. (2014). Nuclear Norm based Matrix Regression with Applications to Face Recognition with Occlusion and Illumination Changes.

[48] Jiang Y., Wu Y., Li W., Wang L., Liao Q. Log-domain polynomial filters for illumination-robust face recognition.

[49] Jia Q., Fang C., Wen D., Ding X. Generating face images under multiple illuminations based on a single front-lighted sample without 3D models.

[50] Nabney I.T. (2004). NETLAB: Algorithms for Pattern Recognition.

[51] Vedaldi A., Fulkerson B. VLFeat: An open and portable library of computer vision algorithms.

[52] Miao Z., Jiang X. (2013). Interest point detection using rank order LoG filter. Pattern Recognit..

[53] Ross A., Rattani A., Tistarelli M. Exploiting the “doddington zoo” effect in biometric fusion.

